# Determining minimum numbers of di-allelic diagnostic markers required to identify introgressions in diploid cross-species hybrid individuals from different types of inter- and backcross populations

**DOI:** 10.1590/1678-4685-GMB-2019-0324

**Published:** 2020-08-21

**Authors:** Joseane Padilha da Silva, Alexandre Rodrigues Caetano

**Affiliations:** ^1^ Embrapa Recursos Genéticos e Biotecnologia, Parque Estação Biológica, Brasília, DF, Brazil

**Keywords:** Power test, hybrid identification, cross-species hybridization, broodstock management

## Abstract

Cross-species hybridizations have been extensively used to generate animals and plants better suited for draft and food and fiber production since Roman times, and are still important in current agricultural practices with growing uses especially in aquaculture. Diagnostic tools based on marker panels with sufficient numbers of markers for accurate identification of cross-species hybrid individuals from intercrossed and backcrossed populations are increasingly necessary for practical, accurate species-purity certification and management of commercial broodstocks. Minimal numbers of di-allelic markers with species-specific alleles required to accurately identify hybrid individuals in intercrossed and advanced backcrossed populations were estimated using power analysis, and ranged from 5 to 191 (α = .05), and from 7 to 293 (α = .01), considering backcross 1 (BC1) to BC6 populations, respectively. Numbers of markers required for accurate hybrid identification observed in simulated BC1 to BC6 populations ranged from 5 to 1,131 and 7 to 8,065, considering error rates ≤ 5% and ≤ 1%, respectively. Estimated and observed numbers of diagnostic markers required for accurate hybrid identification up to four generations of backcrossing fall within practical operational limits of most commercial platforms currently available for genotyping low-density SNP marker panels. Therefore, cost-effective assay panels could be developed to provide practical tools for accurate species-purity certification.

## Introduction

Artificial production of cross-species hybrids has been extensively used to generate animals and plants better suited for a diversity of uses such as draft and production of food and fiber since ancient Roman times (Adams *et al.*, 2007), and still plays an important role in current agricultural practices, with growing use especially in aquaculture. Cross-species hybrids of cultured fish are widely used (reviewed by Bartley *et al.*, 2001) and account for a significant share of current finfish production. F1 hybrids are expected to perform better in captive production systems in respect to productivity and quality traits, as a consequence of the resulting positive heterosis, in spite of the lack of solid studies in many instances to accurately contrast production performances of hybrid populations with parental species (reviewed by Hashimoto *et al.*, 2012).

Several fish hybrids produced in captivity have been shown to be fertile and can naturally generate progeny in both intercrosses with other hybrids and backcrosses with parental species (Bartley *et al.*, 2001), posing a threat to wild populations which co-inhabit river basins where hybrid aquaculture escapees can freely mate with parental species naturally isolated by non-genetic barriers (Hashimoto *et al.*, 2012). Moreover, considering the lack of morphological differences between post-F1 hybrids and parental species reported in many instances (Hashimoto *et al.*, 2011), one of the main challenges found in the large-scale use of hybridization is the potential contamination of pure parental broodstocks (Mair, 2007). Once unknown introgressions have occurred in captive broodstocks, expected results from genetic improvement programs established to breed fish with better productivity and quality traits may become unknowingly compromised, especially because observed high fecundity rates can act to rapidly disseminate introgressed germplasm.

Methods developed to infer population structure and assign individuals to populations using multilocus genotype data (Pritchard *et al.*, 2000) are well established and have been used to assign individuals to populations, identify migrants and admixed individuals, in natural and captive populations, but may not be best suited for practical identification of advanced hybrids in routine testing. Molecular tools based on species-specific diagnostic alleles assayed by PCR-RFLP have been developed for identifying neotropical fish hybrids used for aquaculture (Hashimoto *et al.*, 2010, 2011, 2012; Prado *et al.*, 2011). These tools have been based on low numbers of unlinked markers (up to three) and have been shown to be fully effective in identifying F1 hybrids. However, adequate statistical frameworks to determine numbers of markers required to reliably identify hybrids in advanced intercrossed and backcrossed populations have not been developed and are essential for establishing optimal parameters for designing low-cost diagnostic marker panels based on proper numbers of markers for accurate species-purity certification of individuals and management of commercial broodstocks, and monitoring of wild populations.

Current molecular tools for parentage verification, genetic-disease diagnostics, and genetic improvement of species used for food production are mostly based on SNP markers, as recent technologies have allowed for development of assay platforms that can genotype up to hundreds of thousands of markers in parallel, in highly automated processes which result in low costs and low error rates (Vignal *et al.*, 2002). Basic statistical groundwork and formulae have been stablished to determine minimal numbers of unlinked di-allelic SNP markers with adequate inference power required for parentage verification (Baruch and Weller, 2008) and product tracking (Heaton *et al.*, 2005) in farm animals, using approaches that allow decision making based on power analysis with minimal probabilities of errors. Work presented herein was performed to establish a basic statistical framework to compute minimal numbers of SNP markers with diagnostic (species-specific) alleles required to accurately identify, in routine testing, cross-species hybrid individuals from different types of inter- and backcrossed populations of diploid organisms, based on power analysis, and to verify these in simulated populations.

## Material and Methods

### Determining minimal numbers of markers required for hybrid detection based on power analysis

The established statistical framework is based on the use of bi-allelic SNP markers with species-specific alleles. Therefore, background work is required to identify cross-species SNPs, verified to be fixed for alternative alleles across large numbers of individual samples across different populations of a target species and one or more non-target species used for production of hybrids. Therefore, a target species needs to be shown to carry only a particular allele (f(1)=1, f(2)=0), while other species used for hybridization have to be shown to carry only the alternative allele (f(1)=0, f(2)=1), at any useful SNP marker. Consequently, an F1 cross-species hybrids with the target species is expected to be heterozygous at all diagnostic SNPs (F(12)=1; f(1)=f(2)=0.5). A backcross of an F1 with the target species (BC1) results in allelic and genotypic frequencies as follows: f(1)=0.75, f(2)=0.25; F(11)=0.5, F(12)=0.5, respectively. Table 1 shows expected allelic and genotypic frequencies for different types of inter (F1 and F2) and backcrossed (BC1 to BC6) hybrid populations from distinct, diploid, allogamous species, capable of generating fully fertile hybrid progeny, assuming random mattings and zero mutation, migration, selection and drift at every generation. For a given backcross level, the expected probability of observing the alternative allele at any diagnostic locus is *p*_0_ (or *p* under *H*_0_, Table 1).

**Table 1:** Expected allelic and genotypic frequencies in different intercross and backcross populations between a target and a non-target species and respective hypothesis for statistical tests for hybrid identification.

Population	Expected Allelic Frequencies f(1):f(2)	Expected Genotypic Frequencies F(11):F(12):F(22)	Hypothesis
Target Species	1.0:0.0	1:0:0	-
F1	0.5:0.5	0:1:0	-
F2	0.5:0.5	0.25:0.5:0.25	H_0_: *p*=0.75; H_1_: *p*<0.75
BC1	0.75:0.25	0.5:0.5:0	H_0_: *p*=0.5; H_1_: *p*<0.5
BC2	0.875:0.125	0.75:0.25:0	H_0_: *p*=0.25; H_1_: *p*
BC3	0.9375:0.0625	0.875:0.125:0	H_0_: *p*=0.125; H_1_: *p*<0.125
BC4	0.96875:0.03125	0.9375:0.0625:0	H_0_: *p*=0.0625; H_1_: *p*<0.0625
BC5	0.984375:0.015625	0.96875:0.03125:0	H_0_: *p*=0.03125; H_1_: *p*<0.03125
BC6	0.9921875:0.0078125	0.984375:0.015625:0	H_0_: *p*=0.015625; H_1_: *p*<0.015625

Considering *m* independent loci, the number of occurrences of alternative alleles at diagnostic positions in the genome of an individual of a given backcross level follows a binomial distribution *X* ~ *B*(*m*, *p*_0_). A true hybrid is not identified if X=0, therefore it is necessary to keep *P*(*X* = 0) below a certain level β for accurate identification of true hybrids, that is:

$$\begin{array}{l} P\left(x=0\right)<\text{β}\\ =\left(\begin{array}{c} m\\ 0 \end{array}\right)p_{0}^{0}{\left(1-p_{0}\right)}^{m}<\text{β}\\ ={\left(1-p_{0}\right)}^{m}<\text{β}, \end{array}$$

as a result,

(1) $$m>\frac{\ln\left(\text{β}\right)}{\ln\left(1-p_{0}\right)}$$

represents the minimal number of *m* markers required for accurate identification of true hybrids, which can be determined by calculating the probability of observing an allele from a non-target species after n generations of backcrossing with a target species, as *p* approaches zero. The correct identification of hybrid individuals occurs when a non-target allele is observed at any diagnostic SNP.

### Simulations

Expected numbers of bi-allelic markers required to identify hybrids in all different intercross and backcross populations between a target and a non-target species considered were compared with observed numbers of markers based on simulated populations. R/qtl (Broman *et al.*, 2003) was used to simulate SNP marker positions and genotypes for each evaluated population type based on a genetic map (Nunes *et al.*, 2017) available for Tambaqui (*Colossoma macropomum*), a neotropical fish species with captive production in rapid expansion, commonly hybridized with closely related species (Hashimoto *et al.*, 2011). A total of 7,192 simulated diagnostic SNPs distributed accordingly, with varying marker numbers and positions for each chromosome (n=X=27), were generated in a map with total length of 2,811.2 cM. Populations with different segregation structures (F2, BC1-6) and expected frequencies of diagnostic markers (Table 1), each containing 10,000 individuals, were subsequently generated. A subset of 486 diagnostic markers with a minimal distance of 5cM were selected and subsequently used for hybrid identification in all simulated populations.

**Figure 1:**
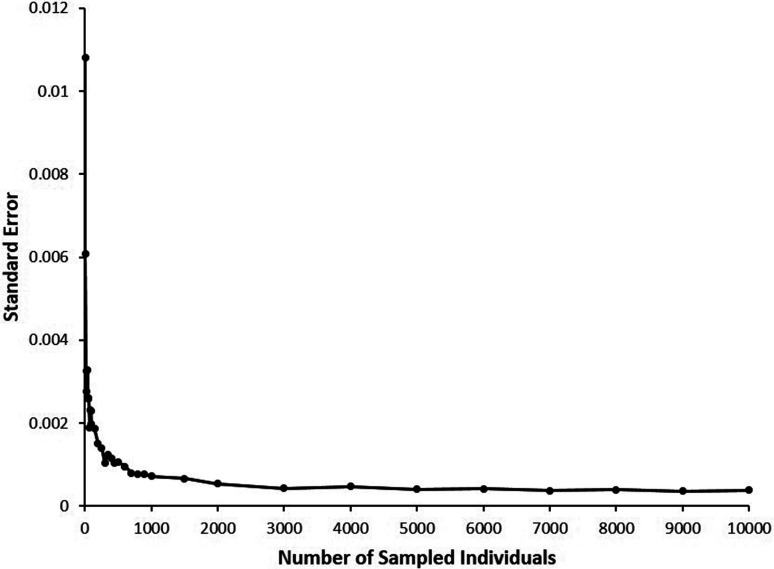
Standard errors for correct identification of hybrid individuals in a simulated population after six generations of backcrossing (BC6) with a target species, obtained using different numbers of sampled individuals from a simulated population with 10,000 individuals.

The most extreme scenario expected in current commercial Tambaqui broodstock populations, represented by a sixth backcross (BC6) generation (F(12) = *p* = 0.015625), was used to determine minimal sampling size required for accurately estimating error rates of hybrid identification in each of the tested populations. A total of 100 random samplings, with replacement, of each considered size (10-100 individuals, in increments of 10; 100-500, in increments of 50; 500-1,000, in increments of 100; 1500; 2,000-10,000 in increments of 1,000) were performed, and rates of correct hybrid identification and respective standard errors determined, based on a total of 486 genotyped diagnostic markers selected. The estimated point where the curve reached an optimal plateau, considering a linear-plateau segmented regression model, was used to determine the minimal number of individuals to be sampled from each population type for proper comparisons with calculated error rates for hybrid identification (Figure 1).

Genotypes from 486 SNP markers from 100 repeated samplings of 300 individuals from each simulated hybrid population (F2, BC1-6) were used for identification of hybrids and computation of error rates. Following the initial sampling and computations, 27 markers were randomly removed (one marker per chromosome), individuals were randomly sampled again and error rates computed. This procedure was repeated for 13 additional rounds, followed by 26 rounds where a single maker was removed per round. Therefore, the total number of markers used for computation of error rates varied from 486 and 1 in each simulated population type. Non-linear equations were derived for BC5 and BC6 observed error rate curves.

## Results

### Estimated minimal number of markers required for hybrid detection based on power analysis

**Figure 2:**
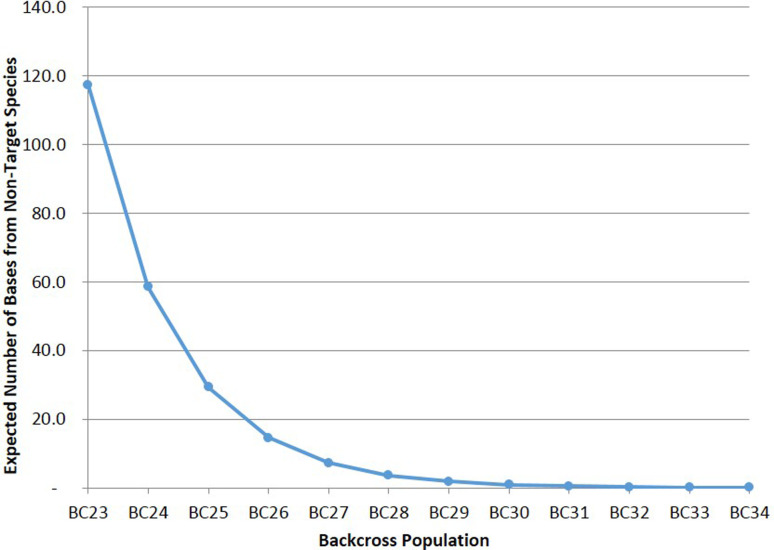
Expected number of base-pairs (E) from a non-target species, considering number of backcross generation with a target species with a genome of 1.2Gbp.

**Figure 3:**
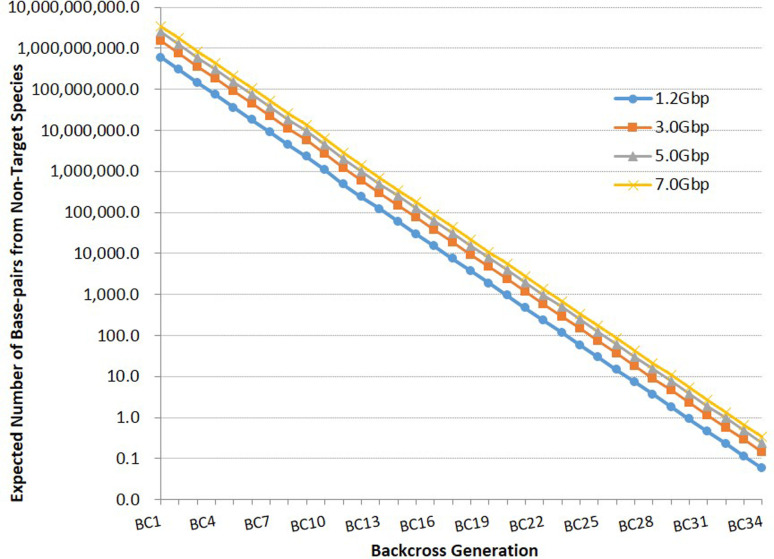
Expected number of base-pairs from a non-target species considering number of backcross generation with a target species with genome size varying from 1.2 to 7.0Gbp.

The critical value for *p* used for calculating expected numbers of independent bi-allelic markers required to identify hybrids in different intercross and backcross populations of a target species with a genome size of 1.2Gbp was determined to be 7.62939E-10 (Figure 1). An extreme situation was considered where every nucleotide could be considered a potential diagnostic marker, and therefore 30 generations of backcrossing an F1 hybrid would be required for the expected number of base-pairs from a non-target species to be <1bp. Figure 2 shows expected numbers of base-pairs from a non-target species across backcross generations 1 to 34 for a species with a genome with 1.2Gbp. The relationship of genome size with number of base-pairs from a non-target species expected considering number of backcross generation, is shown on Figure 3.

**Figure 4:**
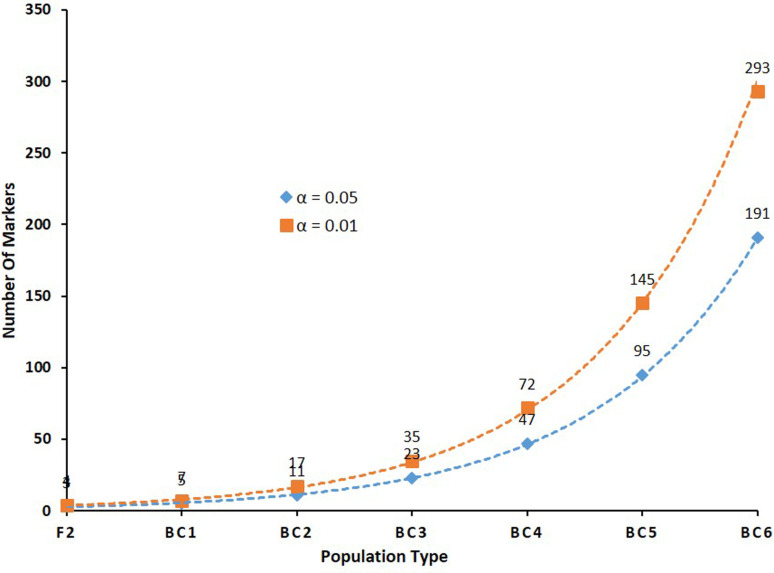
Estimated numbers of independent bi-allelic markers expected for correctly identifying hybrid individuals in different intercross and backcross populations between a target and a non-target species based on power analysis, considering *p* = 7.62939E-10, Power(1 - β) ≅ 99%, and α = .05 *or* .01.

Table 2 shows the estimated numbers of diagnostic independent bi-allelic markers required to identify hybrids in different intercross and backcross populations between a target and a non-target species based on power analysis, considering *p* = 7.62939E-10, with Power(1 - β) ≅ 99%, as in equation 1. Estimated numbers of required markers ranged from 5 to 191 (α = .05), and from 7 to 293 (α = .01), considering populations BC1 to BC6, respectively (Figure 4).

**Table 2:** – Estimated numbers of bi-allelic markers required to identify hybrid individuals in different intercross and backcross populations between a target and a non-target species based on power analysis, considering *p* = 7.62939E-10 and Power (1 - β) ≅ 99%, and observed on simulated populations, respectively.

Population Type	Estimated numbers based on power analysis		Observed numbers based on simulated populations	
	α = .05	α = .01	Error Rate ≤ 5%	Error Rate ≤ 1%
F2	3	4	3	4
BC1	5	7	5	7
BC2	11	17	11	17
BC3	23	35	23	54
BC4	47	72	54	135
BC5	95	145	189	1,616^*^
BC6	191	293	1,131^*^	8,065^*^

**Figure 5:**
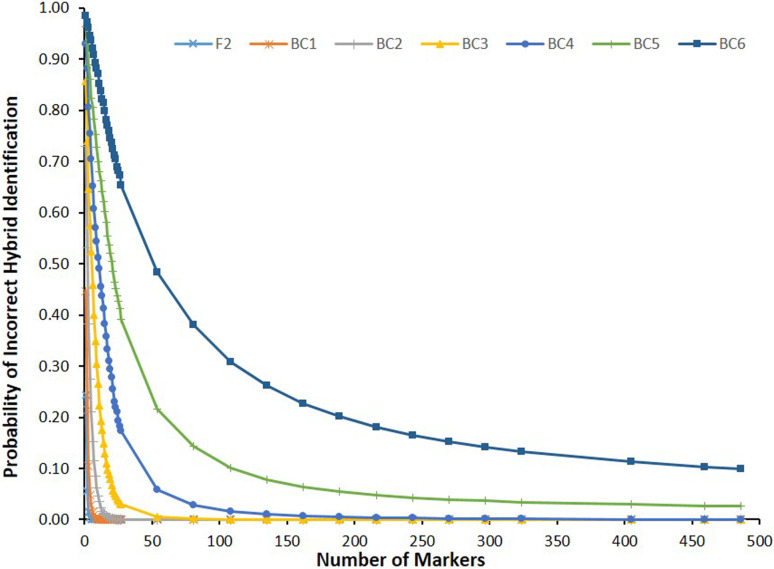
Probability of incorrect identification of hybrid individuals observed in different types of simulated inter and backcross populations, considering increasing numbers of diagnostic markers.

* Calculated using non-linear equations derived from observed error rate curves in simulated populations (Figure 5)

### Simulations

Figure 1 shows the relationship between number of sampled individuals and standard error rates for hybrid identification in the most extreme intercrossed population considered (BC6). The estimated point where the curve reaches an optimal plateau was 258.716 ± 41.983 individuals, considering a linear-plateau segmented regression model, which represents the minimal number of individuals that should be sampled for accurate calculation of error rates for hybrid identification. Subsequently, 100 independent samples of 300 simulated individuals were checked for correct hybrid identification, for accurate calculation of error rates in all remaining population types and numbers of diagnostic markers considered.

**Figure 6:**
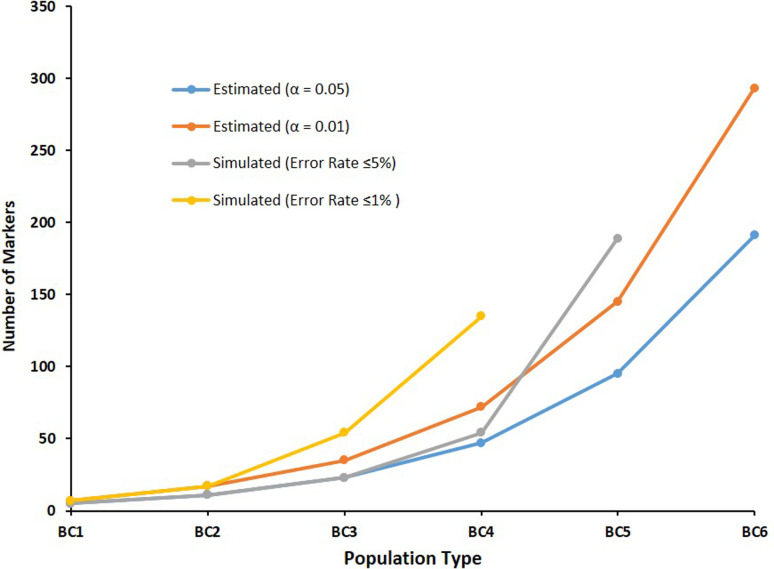
Comparisons of estimated numbers of independent bi-allelic markers required to identify hybrid individuals in different intercross and backcross populations based on power analysis and observed error rates in simulated populations.

Observed error rates for correct hybrid identification in all simulated types of inter and backcross populations using from 1 to 486 markers are shown in Table S1. Figure 5 shows observed error rate curves observed for each simulated population. Observed numbers of markers required to identify hybrids in simulated populations (Table 2) were compared with expected numbers (Figure 6). Non-linear equations derived for BC5 (*Y* ~ 6.36973 + 4.51791*X*_−1.27593_,R^2^ = 99.5%) and BC6 (*Y* ~ -26.0083 + 30.96739*X* ^−1.20855^,*R* ^2^ = 99.9%), were used to calculate numbers of markers (*Y*) required for accurate hybrid identification with error rate *X* in BC5 and BC6 (Table 2). Critical command lines used in simulations with R/qtl are provided in File S1.

## Discussion

Proper validation of molecular diagnostic tools for identification of cross-species hybrid individuals requires adequate statistical tests to determine expected error rates and confidence levels associated with the number and type of markers used, considering respective analytical limits associated with admixture and introgression levels in target populations, in addition to other innate error sources (i.e. genotyping errors). Numbers of required markers for correct hybrid identification estimated and simulated herein show that a minimum of four or seven independently-segregating nuclear bi-allelic markers with species-specific alleles are required for accurate individual hybrid identification in F2 and BC1 populations, respectively, considering false positive (α) error rates <1%. Conversely, an estimated minimum of up to 95 and 145 markers are required for accurate hybrid identification, considering α of 5% and 1%, respectively, when introgression levels considered are down to 1.56%, which is expected in a BC5.

Proposed tests for identification of Siluriforme hybrids (*Pseudoplatystoma corruscans* and *P. reticulatum*) have been based on a single nuclear and a single mitochondrial marker with species-specific alleles (Prado *et al.*, 2011), or on eight microsatellite markers with differing allele frequencies with no exclusive species-specific alleles (Carvalho *et al.*, 2013). Similarly, a molecular test for identification of Serrasalmid hybrids (*Colossoma macropomum, Piaractus mesopotamicus and P. brachypomus*) was proposed based on two nuclear markers and two mitochondrial markers (Hashimoto *et al.*, 2011). In both groups of species, mitochondrial markers are haploid and inherited maternally. These examples include species frequently hybridized in commercial aquaculture operations for production of fry destined to grow out and human consumption, which generate fully fertile hybrids that can be readily intercrossed or backcrossed to pure parental species. Obtained estimates and observations in simulated populations herein show that significantly higher numbers of independently segregating markers with species-specific alleles are required for accurate identification of advanced-cross hybrids and proper certification and management of pure species broodstocks, than may be currently under use considering existing proposed tests. Diagnostic tests based on 2-3 markers with species-specific alleles may be appropriate for identification of F1 crossbreds but, considering observed results, are far from having sufficient analytical power for identifying introgressions which may eventually have negative impacts on genetic improvement programs and conservation of natural populations.

Methods using allozyme and microsatellite data to identify hybrids and introgressions based on allele frequency differences between populations have been applied to identify F1, F2 and backcross 1 (BC1) hybrids in simulated data from wild brown trout (*Salmo truta*) populations from areas stocked with hatchery fish (Sanz *et al.*, 2009). In addition, studies based on population allele frequency differences using SNP data derived from restriction-associated DNA (RAD-Seq) sequencing (Baird *et al.*, 2008) have identified hybrids between two closely related sole species (Souissi *et al.*, 2018), and European (*Anguilla anguilla*) and American (*A. rostrata*) eels (Pujolar *et al.*, 2014). Even though the cited methods clearly identified the occurrence of hybridization events, hybrid individuals beyond the F2 or BC1 levels could not be accurately identified, and therefore such methods could not be used for routine diagnostics and certification of individual fish in situations where broodstock have been kept captive for several generations.

Listed formulae and performed simulations considered a number of critical assumptions, including uniform distribution and independent segregation of markers, marker neutrality, and Hardy–Weinberg equilibrium in advanced intercrossed and backcrossed hybrid populations, as have other similar studies performed to estimate exclusion probabilities in random mating and similar types of structured populations (Jamieson and Taylor, 1997; Saunders *et al.*, 2007; Baruch and Weller, 2008; Carvalho *et al.*, 2013). Considering the proposed framework is based on the use of extensively validated markers, shown to have species-specific alleles, Type I errors error rates (α) associated with not identifying a true hybrid are expected to result from genotyping errors, *de novo* mutations, and other issues addressed subsequently, which may result in a lack of assayed markers in genome regions introgressed from non-target species.

Deviations between estimated and observed numbers of markers required for correct identification of hybrids in simulated populations were observed in advanced backcross populations (Table 2, Figure 6). Calculations of expected numbers of markers considered complete linkage independence between diagnostic markers. However, considering the number of effective independent markers is finite, observed differences between estimated and observed numbers of required markers can be attributed to the lack of independent assortment between markers with distances <50cM. In the particular simulated species n=X=27, and deviations between calculated and observed numbers of makers required for accurate hybrid identification were observed to emerge when *m* > 27. Moreover, resulting residual genome fragments from a non-target species in advanced backcrosses may be distributed in increasingly larger numbers of smaller chromosome fragments, with diminishing probabilities of presence of at least one diagnostic marker, as a consequence of subsequent recombination events across generations, and may be affected by species-specific recombination rates. Consequently, deviations between calculated and observed numbers of markers required for accurate hybrid identification may be even higher than expected because of lack of independence, which may explain the observed 27-fold difference between expected and observed numbers of markers required for accurate hybrid identification in a BC6 (α < .01). Therefore, final determination of minimal numbers of diagnostic markers required for species-purity certification should consider chromosome number, average recombination rate and supposed number of generations when earliest introgressions may have occurred in target populations.

Calculations of test power require the use of critical non-zero values for *p*, as *p* approaches zero, for solving equation (1). Instead of using a random ~0 value for *p*, an actual value was calculated considering the expected number of generations of backcrossing an F1 hybrid that would be required to remove every nucleotide from a non-target species from a genome with 1.2Gbp (30 generations). Critical non-zero values for *p* calculated for genomes up to 7.0Gbp (Figure 2) were similarly observed after 33 generations of backcrossing, therefore indicating that genome size will have little effect on numbers of markers required for accurate hybrid identification. Calculations of marker numbers with larger *p* values (>.0001) were performed (data not shown) but did not affect observed results.

A segmented regression model was applied to determine minimal sampling size required for accurate estimation of error rates for hybrid identification in different types of simulated populations (Figure 3). Based on this finding, multiple samplings of 300 individuals from each tested population were therefore considered to be sufficient for accurate calculation of error rates in all studied populations. Simulations were limited to a maximum of 486 markers (18 per chromosome) as initial calculations indicated <300 markers would provide sufficient statistical power for accurate hybrid identification even in BC6 populations (α = .01). Unwanted introgressions in captive populations are likely to follow complex admixture patterns, similarly to natural populations. However, allele frequencies in late-generation hybrid populations resulting from these processes are likely to resemble those expected in advanced backcrossed populations. Considering generation intervals in these particular species are 3-4 years, and that most of current commercial broodstocks are descendants of animals captured in nature since year 2000 (Hashimoto *et al.*, 2012), the most extreme possible case currently expected of an advanced-cross hybrid would be a BC5, falling within the limit of the most advanced backcross population (BC6) used for all calculations and simulations.

Estimated and observed numbers of diagnostic markers required for accurate hybrid identification up to four generations of backcrossing (BC4) fall well within practical operational limits of most commercial platforms currently available for genotyping low density SNP marker panels. Therefore, cost-effective assay panels could be developed to provide practical tools for accurate species-purity certification of individuals and management of commercial broodstocks, and monitoring of wild populations. In addition, proper numbers of diagnostic markers may be included within medium (tens of thousands) and high-density (hundreds of thousands) marker panels already available or under development for applications such as genome-wide genetic evaluations and selection (Tsai *et al.*, 2015). Additional strategies using data from multiple adjacent SNPs generated with medium and high-density panels have also been developed to identify population-specific haplotypes (Halbert *et al.*, 2005; Simcic *et al.*, 2015), which are particularly useful when exclusive SNP variants are not available, and that can in turn be used to identify admixed individuals and eventually reconstitute original genetic backgrounds, using specifically designed breeding strategies. Reported genotyping errors for SNP markers vary according to platform and can be as low as 0.1% (Saunders *et al.*, 2007). Considering that certifying species purity at the BC4 level will require >100 genotyped markers, requiring at least two conflicts to classify an individual as a hybrid may be justified, even if genotyping error rates are as low as cited by technology providers.

The methodology described herein may also be applied to determine minimal numbers of diagnostic markers required for identification of crossbreds derived from intercrossing different breeds/populations within a species. Tools for breed allocation for establishment of genetic resource conservation populations (Negrini *et al.*, 2007) and certification of animal-derived food products (Sasazaki *et al.*, 2007) have been developed, considering minimal to zero levels of inter or backcrossing of subjects. As shown, the high correlation observed between admixture level and expected numbers of diagnostic markers required to identify hybrids should be considered in cases where low error rates can be accepted in identifying subjects with low levels of introgressions from other populations/breeds.

## Conclusions

The obtained results established a statistical ground work for performing molecular diagnostic tests to identify individuals generated from undesired cross-species hybridizations with respective confidence levels, in different types of inter and backcross populations. Estimated and observed numbers of diagnostic markers required for proper individual hybrid identification up to four generations of backcrossing fall well within practical operational limits of most commercial platforms currently available for genotyping low-density SNP marker panels. Therefore, cost-effective assay panels could be developed to provide practical tools for accurate routine species-purity certification and management of commercial broodstocks and monitoring of wild populations.

## Supplementary material

The following online material is available for this article:

Table S1 Probabilities of error in identifying a true individual hybrid with different numbers of di-allelic markers with species-specific alleles observed in different types of simulated populations.

File S1 Critical parameters used in simulations with R/qtl.
