# Supplementary material for: Determining minimum numbers of di-allelic diagnostic markers required to identify introgressions in diploid cross-species hybrid individuals from different types of inter- and backcross populations
Source: Genet Mol Biol. 2020 Aug 21;43(3):e20190324. doi: 10.1590/1678-4685-GMB-2019-0324 (PMC7445936; doi:10.1590/1678-4685-GMB-2019-0324)
Supplement: Supplementary file 1 [file 1415-4757-GMB-43-3-e20190324-suppl1.pdf]

Genetics and Molecular Biology

https://doi.org/10.1590/1678-4685-GMB-2019-0324

Supplementary Material to "Determining minimum numbers of di-allelic diagnostic markers required to identify introgressions in diploid cross-species hybrid individuals from different types of inter and backcross populations"

Table S1 – Probabilities of error in identifying a true individual hybrid with different numbers of di-allelic markers with species-specific alleles observed in different types of simulated populations.

			**Simulated Population Type**													
**Diagnostic Markers**	**Chromosomes with markers**	**Markers per Chromosome**	**F2**		**BC1**		**BC2**		**BC3**		**BC4**		**BC5**		**BC6**	
Number (N)			**P.E.**	**S.E.**	**P.E.**	**S.E.**	**P.E.**	**S.E.**	**P.E.**	**S.E.**	**P.E.**	**S.E.**	**P.E.**	**S.E.**	**P.E.**	**S.E.**
1	1	1	0.2433	0.0010	0.4457	0.0015	0.7290	0.0024	0.8578	0.0016	0.9294	0.0009	0.9639	0.0009	0.9852	0.0004
2	2	1	0.0559	0.0010	0.2148	0.0015	0.5321	0.0024	0.7397	0.0018	0.8812	0.0015	0.9313	0.0015	0.9715	0.0008
3	3	1	0.0147	0.0007	0.1042	0.0010	0.3823	0.0019	0.6467	0.0024	0.8062	0.0028	0.8894	0.0016	0.9615	0.0009
4	4	1	0.0038	0.0003	0.0470	0.0009	0.2748	0.0017	0.5755	0.0023	0.7544	0.0029	0.8603	0.0016	0.9465	0.0011
5	5	1	0.0011	0.0002	0.0281	0.0008	0.2099	0.0019	0.5241	0.0024	0.7045	0.0028	0.8242	0.0017	0.9367	0.0012
6	6	1	0.0002	0.0001	0.0146	0.0007	0.1517	0.0019	0.4584	0.0025	0.6519	0.0028	0.8066	0.0019	0.9217	0.0013
7	7	1	0.0000	0.0000	0.0086	0.0005	0.1158	0.0017	0.4006	0.0023	0.6068	0.0028	0.7826	0.0022	0.9091	0.0014
8	8	1	0.0000	0.0000	0.0041	0.0004	0.0853	0.0014	0.3497	0.0024	0.5703	0.0025	0.7525	0.0023	0.8932	0.0014
9	9	1	0.0000	0.0000	0.0021	0.0003	0.0614	0.0011	0.3049	0.0023	0.5443	0.0024	0.7281	0.0023	0.8826	0.0015
10	10	1	0.0000	0.0000	0.0012	0.0002	0.0464	0.0011	0.2657	0.0022	0.5125	0.0025	0.7003	0.0024	0.8719	0.0016
11	11	1	0.0000	0.0000	0.0006	0.0001	0.0345	0.0010	0.2228	0.0019	0.4903	0.0025	0.6804	0.0024	0.8518	0.0018
12	12	1	0.0000	0.0000	0.0002	0.0001	0.0230	0.0008	0.1934	0.0017	0.4554	0.0026	0.6618	0.0024	0.8376	0.0018
13	13	1	0.0000	0.0000	0.0001	0.0001	0.0159	0.0007	0.1749	0.0017	0.4368	0.0027	0.6414	0.0024	0.8224	0.0019
14	14	1	0.0000	0.0000	0.0001	0.0000	0.0122	0.0007	0.1495	0.0017	0.4130	0.0027	0.6218	0.0025	0.8152	0.0019
15	15	1	0.0000	0.0000	0.0000	0.0000	0.0102	0.0006	0.1295	0.0015	0.3823	0.0028	0.6029	0.0025	0.7985	0.0020
16	16	1	0.0000	0.0000	0.0000	0.0000	0.0072	0.0005	0.1102	0.0015	0.3574	0.0029	0.5803	0.0025	0.7803	0.0021
17	17	1	0.0000	0.0000	0.0000	0.0000	0.0058	0.0005	0.0979	0.0014	0.3333	0.0027	0.5537	0.0024	0.7704	0.0021
18	18	1	0.0000	0.0000	0.0000	0.0000	0.0041	0.0004	0.0894	0.0013	0.3106	0.0027	0.5374	0.0024	0.7606	0.0022

19	19	1	0.0000	0.0000	0.0000	0.0000	0.0032	0.0004	0.0788	0.0013	0.2935	0.0026	0.5207	0.0024	0.7465	0.0021
20	20	1	0.0000	0.0000	0.0000	0.0000	0.0026	0.0003	0.0680	0.0013	0.2774	0.0025	0.5040	0.0025	0.7367	0.0021
21	21	1	0.0000	0.0000	0.0000	0.0000	0.0017	0.0003	0.0571	0.0011	0.2549	0.0025	0.4849	0.0024	0.7239	0.0021
22	22	1	0.0000	0.0000	0.0000	0.0000	0.0010	0.0002	0.0513	0.0011	0.2305	0.0026	0.4648	0.0024	0.7122	0.0022
23	23	1	0.0000	0.0000	0.0000	0.0000	0.0007	0.0002	0.0457	0.0010	0.2188	0.0025	0.4513	0.0024	0.7043	0.0022
24	24	1	0.0000	0.0000	0.0000	0.0000	0.0005	0.0001	0.0409	0.0010	0.2109	0.0025	0.4371	0.0023	0.6894	0.0022
25	25	1	0.0000	0.0000	0.0000	0.0000	0.0004	0.0001	0.0377	0.0010	0.1934	0.0024	0.4268	0.0024	0.6828	0.0022
26	26	1	0.0000	0.0000	0.0000	0.0000	0.0003	0.0001	0.0320	0.0009	0.1821	0.0022	0.4126	0.0023	0.6732	0.0022
27	27	1	0.0000	0.0000	0.0000	0.0000	0.0002	0.0001	0.0307	0.0010	0.1733	0.0019	0.3922	0.0021	0.6529	0.0025
54	27	2	0.0000	0.0000	0.0000	0.0000	0.0000	0.0000	0.0054	0.0004	0.0591	0.0013	0.2158	0.0018	0.4830	0.0028
81	27	3	0.0000	0.0000	0.0000	0.0000	0.0000	0.0000	0.0012	0.0002	0.0279	0.0009	0.1428	0.0016	0.3812	0.0025
108	27	4	0.0000	0.0000	0.0000	0.0000	0.0000	0.0000	0.0004	0.0001	0.0155	0.0006	0.1018	0.0012	0.3088	0.0025
135	27	5	0.0000	0.0000	0.0000	0.0000	0.0000	0.0000	0.0001	0.0001	0.0098	0.0005	0.0782	0.0011	0.2613	0.0022
162	27	6	0.0000	0.0000	0.0000	0.0000	0.0000	0.0000	0.0000	0.0000	0.0066	0.0005	0.0633	0.0010	0.2267	0.0019
189	27	7	0.0000	0.0000	0.0000	0.0000	0.0000	0.0000	0.0000	0.0000	0.0048	0.0004	0.0548	0.0009	0.2014	0.0018
216	27	8	0.0000	0.0000	0.0000	0.0000	0.0000	0.0000	0.0000	0.0000	0.0036	0.0003	0.0485	0.0008	0.1805	0.0017
243	27	9	0.0000	0.0000	0.0000	0.0000	0.0000	0.0000	0.0000	0.0000	0.0030	0.0003	0.0432	0.0007	0.1650	0.0017
270	27	10	0.0000	0.0000	0.0000	0.0000	0.0000	0.0000	0.0000	0.0000	0.0023	0.0003	0.0395	0.0007	0.1522	0.0015
297	27	11	0.0000	0.0000	0.0000	0.0000	0.0000	0.0000	0.0000	0.0000	0.0014	0.0002	0.0366	0.0006	0.1416	0.0013
324	27	12	0.0000	0.0000	0.0000	0.0000	0.0000	0.0000	0.0000	0.0000	0.0011	0.0002	0.0343	0.0005	0.1328	0.0012
405	27	15	0.0000	0.0000	0.0000	0.0000	0.0000	0.0000	0.0000	0.0000	0.0005	0.0001	0.0299	0.0005	0.1129	0.0009
459	27	17	0.0000	0.0000	0.0000	0.0000	0.0000	0.0000	0.0000	0.0000	0.0003	0.0001	0.0274	0.0004	0.1030	0.0009
486	27	18	0.0000	0.0000	0.0000	0.0000	0.0000	0.0000	0.0000	0.0000	0.0002	0.0001	0.0269	0.0004	0.0995	0.0009

Yellow and red cells identify the critical point where P.E. reaches values ≤ .05 and ≤ .01, respectively.
