# Supplementary material for: Determining minimum numbers of di-allelic diagnostic markers required to identify introgressions in diploid cross-species hybrid individuals from different types of inter- and backcross populations
Source: Genet Mol Biol. 2020 Aug 21;43(3):e20190324. doi: 10.1590/1678-4685-GMB-2019-0324 (PMC7445936; doi:10.1590/1678-4685-GMB-2019-0324)
Supplement: Supplementary file 2 [file 1415-4757-GMB-43-3-e20190324-suppl2.pdf]

Genetics and Molecular Biology

https://doi.org/10.1590/1678-4685-GMB-2019-0324

Supplementary Material to "Determining minimum numbers of di-allelic diagnostic markers required to identify introgressions in diploid cross-species hybrid individuals from different types of inter and backcross populations"

File S1 – Critical parameters used in simulations with R/qtl.

```
###########################################################################
R/qtl (Broman et al. 2003) was used to simulate SNP marker positions and
genotypes for each evaluated population type based on a genetic map (Nunes et al. 13 2017) available for Tambaqui (Colossoma Macropomum)

@Parameters
Len \\ A vector specifying the chromosome lengths (in cM)
n.mar \\ A vector specifying the number of markers per chromosome
include.x \\ Indicates whether the last chromosome should be considered the X chromosome
eq.spacing \\If FALSE, markers will not be equally spaced.
mapTamb \\ A list whose components are vectors containing the marker locations on each of the chromosomes
n.ind \\ Number of individuals to simulate
type \\ Indicates the Populations segregation structures (F2, BC1-6)
###########################################################################
// Simulates the genetic map
SET sim.map(len=Len.Chr,n.mar=N.mark,include.x=F, eq.spacing=F)
// Simulates the population type
SET sim.cross(mapTamb, n.ind=300,type="pop")

#########################################################################
According to the sim.map and the sim.cross simulation this program computes error rates in two scnenarios: i) when all of 27 chromosomes are visited and, ii) when less than 27 chromosomes are visited.
@Parameters
lengthC \\total of chromosomes
c \\ list of chomosomes
m \\ list of markers
sample \\ list of selected sample
```

```
er \\ list of error rates calculated
newC \\ new list of chomosomes
##########################################################################

//Computes error rates in the scenario all of 27 chromosomes are visited

REPEAT 100times
  While(lengthC=27)
    FOR c=1 TO 27
       FOR m=18 TO 1 DO
      sample= select m markers of the total of lengthC*m  markers from the c chromosome
  FIM_FOR
END_REPEAT
RETURN er[sample]#error rates

//Computes error rates in the scenario where less than 27 chromosomes are visited
REPEAT 100times
  FOR c=1 TO 27 DO
  newC= select a c1 random sample from the total of c    chromosome
  FOR newC = 26 TO 1 DO
    FOR m=26 TO 1 DO
                sample= select 1 marker from the newC chromosome
  FIM_FOR
END_REPEAT
RETURN er[sample]#error rates
```
